# Preparation, characterization, and performance evaluation of UiO-66 analogues as stationary phase in HPLC for the separation of substituted benzenes and polycyclic aromatic hydrocarbons

**DOI:** 10.1371/journal.pone.0178513

**Published:** 2017-06-05

**Authors:** Weiwei Zhao, Chaoyan Zhang, Zengguang Yan, Youya Zhou, Jianrong Li, Yabo Xie, Liping Bai, Lin Jiang, Fasheng Li

**Affiliations:** 1 State Key Laboratory of Environmental Criteria and Risk Assessment, Chinese Research Academy of Environmental Sciences, Beijing, China; 2 Beijing Key Laboratory for Green Catalysis and Separation and College of Environmental and Energy Engineering, Beijing University of Technology, Beijing, China; 3 Beijing Municipal Research Institute of Environmental Protection, Beijing, China; Ludwig-Maximilians-Universitat Munchen, GERMANY

## Abstract

UiO-66 analogues are good candidates as stationary phase in HPLC because of their chemical/thermal stability, large surface area, and two cage structures. Here, two UiO-66 analogues, UiO-66-NH_2_ and UiO-67, were synthesized and used as stationary phase in HPLC to evaluate their performance in the separation of substituted benzenes (SBs) and polycyclic aromatic hydrocarbons (PAHs). The results showed that SBs could be well separated on UiO-66-NH_2_ column but not on UiO-67 column. Nonetheless, PAHs could be well separated on UiO-67 column. The separation mechanisms of SBs and PAHs on UiO-66 analogues may be involved in the pore size and functional group in the frameworks of UiO-66 analogues. Introduction of the–NH_2_ into UiO-66 significantly reduced its adsorption capacity for SB congeners, which resulted in less separation of SBs on UiO-66-NH_2_. As for the separation of PAHs on UiO-67 column, the π-π stacking effect was supposed to play a vital role.

## 1. Introduction

Metal-organic frameworks (MOFs) are a category of complexes constructed by organic ligands and inorganic nodes [1]. Most MOFs have infinite network structures and specific properties, such as large surface area, high adsorption affinity, accessible cages, tunable pore dimension, and excellent thermal stability [2,3]. Consequently, MOFs have aroused increasing research interests for their potential application in many fields [4–7]. Particularly, because of their pore dimension tunability and in-pore functionalization without topological change, MOFs endow great potential for application in analytical chemistry [8]. Recently, several MOF materials, including MOF-5, MIL-101(Cr), MIL-47, ZIF-8, and HKUST-1, have been tentatively applied in chromatography [9–13]. Especially, due to the considerable difference of MOFs in topological structure, the effects of pore sizes in MOFs on chromatographic separation have been intensively studied [13, 14]. In most cases, excellent separation was achieved based on a combination of molecular sieving and adsorption effects. However, due to their potential instability in most solvents, MOFs were only limitedly used in high performance liquid chromatography (HPLC) [15, 16].

UiO-66 is Zr-cluster-based MOF consisting of cationic Zr_6_O_4_(OH)_4_ nodes and organic linkers. Due to the combination of strong Zr-O bonds, the UiO-66 possesses high thermal stability and broad solvent resistance [15]. Because of these physicochemical properties, UiO-66 has been used in chromatographic separation process [14, 17]. In our previous study, UiO-66 was slurry-packed in a chromatographic column and used as stationary phase to separate substituted benzenes (SBs) and polycyclic aromatic hydrocarbons (PAHs) in NP- and RP-HPLC [17]. However, the mechanism for the separation of SBs or PAHs by UiO-66 as well as the structure-performance relationship of UiO-66 analogues remains little understood. In the present study, two UiO-66 analogues, UiO-66-NH_2_ and UiO-67, were used as stationary phase in HPLC to separate SBs and PAH, and the structure-performance relationship of UiO-66 analogues was assessed.

The amino-functionalized metal-organic framework UiO-66-NH_2_ and the linker-lengthened metal-organic framework UiO-67 are isoreticular MOFs both derived from UiO-66. The UiO-66-NH_2_ was obtained by replacing the UiO-66 organic linker H_2_BDC with amino-functionalized linker H_2_BDC-NH_2_, and the UiO-67 was obtained by replacing H_2_BDC with longer linker H_2_BPDC [18,19]. The presence of -NH_2_ in UiO-66-NH_2_ endows it with some special properties and further results in some potential application, such as photocatalysis in oxidation/reduction processes and adsorption of nitrogen-containing compounds [20–26]. While considering the high surface area, big pore volume and large pore size of UiO-67 due to its long linker, UiO-67 was mostly used in adsorption of small molecules, such as CO_2_, CH_4_, and H_2_ [27–29]. Both UiO-66-NH_2_ and UiO-67 have similar topological framework, which is also similar to that of UiO-66. For example, the UiO-66, UiO-66-NH_2_ and UiO-67 have the same metal coordination, similar secondary building unit (SBU), and similar interlinking mode of SBUs. The three MOFs have a certain solvent stability property, which is essential for the potential application in HPLC separation. However, the UiO-66, UiO-67 and UiO-66-NH_2_ has different pore size from each other, which enables an investigation of the effects of pore size on the performance of MOFs in chromatographic separation. In the present study, UiO-66-NH_2_ was applied for NP- and RP-HPLC separation, whereas UiO-67 was used only for NP-HPLC separation due to its lower solvent stability. What’s more, the structure-performance relationship of UiO-66 analogues in the same topology structures was demonstrated.

## 2. Materials and methods

### 2.1. Chemicals and reagents

All chemicals and reagents were of high purity (analytical grade or higher). Zirconium chloride (ZrCl_4_), terephthalic acid (H_2_BDC), 2-amino-terephthalic acid (H_2_BDC-NH_2_), 4,4ˊ-biphenyldicarboxylic acid (H_2_PDC), N,N-dimethylformamide (DMF), concentrated hydrochloric acid (HCL), ethanol, n-hexane, and benzoic acid were all analytical grade and purchased from Sinopharm Chemical Reagent Co., Ltd. (Shanghai, China). *O-*xylene, *m-*xylene, *p-*xylene, ethylbenzene, styrene, naphthalene, fluorene, acenaphthene, anthracene, phenanthrene, pyrene, chrysene, phenol and cresol were all analytical grade and obtained from TCI Development Co., Ltd. (Shanghai, China). Methanol (MeOH), acetonitrile (CH_3_CN), n-hexane, and methylene chloride (DCM) were chromatographic grade and purchased from J&K Scientific Ltd. (Beijing, China). Ultrapure water was obtained from Milli-Q Water Purification System (Merck Millipore, Germany).

### 2.2. Instrumentations

The powder X-ray diffraction (PXRD) patterns of UiO-66 analogues were obtained on a BRUKER D8-Focus Bragg-Brentano X-ray Powder Diffractometer equipped with a Cu sealed tube (*λ* = 1.54178 nm) at room temperature. Thermogravimetric analysis (TGA) was performed on a TGA-50 (SHIMADZU) thermogravimetric analyzer with a heating rate of 2°C·min^–1^ under N_2_ atmosphere. The N_2_ adsorption isotherms were measured on a Micromeritics ASAP2020 surface area and pore analyzer. The scanning electron microscopy (SEM) images were recorded on a Shimadzu SS-550 scanning electron microscope at 15.0 kV.

The MOFs-packed columns were prepared using a HY-HPLC-M chromatographic column packing equipment (Singapore Hydratech Industries Pie Ltd., Beijing, China). HPLC separations were performed on a LC-20AT chromatograph equipped with a tunable UV-Vis detector, an auto-sampler, a binary pump, a column oven, and a LC-solution chromatography data system (Shimadzu, Kyoto, Japan).

### 2.3. Synthesis and activation of UiO-66 analogues

UiO-66 was synthesized and activated following to the methods described in our previous study [17]. Briefly, 0.932 g of ZrCl_4_, 1.32 g of terephthalic acid, 0.665 mL of concentrated hydrochloric acid, and 24 mL of DMF were mixed in a Teflon-lined bomb. After ultrasonic treating for 5 min, the bomb was sealed and then heated at 120^°^C for 16 h. The white solid product was obtained after cooling down and centrifuging at 8000 rpm for 10 min. The product was washed three times sequentially with DMF and acetone, and then dried at 60^°^C.

UiO-66-NH_2_ was synthesized in the same way as that of UiO-66 but replacing H_2_BDC with H_2_BDC-NH_2_, according to the method described by Zhang et al. [30]. The product UiO-66-NH_2_ was activated using solvent exchange method (washed with DMF and acetone in turn). UiO-67 was synthesized by adding 0.12 g of ZrCl_4_, 0.125 g of H_2_PDC, and 1.83 g of H_2_BDC into 20 mL of DMF in a glass vial. The vial was sealed and then heated at 100°C for two days. The resultant white powder was collected and washed with DMF and acetone in sequence for three times, and then dried at 80°C.

### 2.4. Preparation of packed columns for HPLC analysis

The UiO-66 packed column for RP- and NP-HPLC separation was prepared following the method as described in our previous study [17]. For the preparation of UiO-66-NH_2_ packed column, 1.20 g of UiO-66-NH_2_ was dispersed in 50 mL n-hexane under ultrasonication for 5 min. The resultant suspension was then downward packed into a stainless steel column (5 cm long, 4.6 mm i.d.) under 5000 psi for 15 min. The suspension was kept intact by the stainless steel frit with a pore size of 2 μm. The packed column used for NP-HPLC was conditioned with n-hexane at a flow of 1.0 mL·min^−1^ for 1 h. The packed column used for RP-HPLC was conditioned with methanol at a flow of 0.5 mL·min^−1^ for 1 h. The UiO-67 packed column was prepared as follows: the well-dispersed suspension of 1.30 g of UiO-67 in 50 mL n-hexane was downward packed into a stainless steel column (5 cm long, 4.6 mm i.d.) under 5000 psi for 15 min. The UiO-67 packed column was assembled in a similar way to that of the preparation of UiO-66-NH_2_ packed column except that1.3 g of UiO-67 rather than 1.2 g of UiO-66-NH_2_ was used.

### 2.5. HPLC separation experiments

The prepared packed column was connected to the HPLC system to start the HPLC separation experiments under the indicated chromatographic conditions. The RP-HPLC separations using UiO-66-NH_2_ as the stationary phase were performed using MeOH/H_2_O as mobile phase at a flow rate of 0.5 mL·min^−1^, with UV detection at 254 nm and column temperature at 30°C. The NP-HPLC separation experiments were conducted using UiO-66-NH_2_ and UiO-67 packed columns, with n-hexane/DCM as mobile phase at a flow rate 1.0 mL·min^−1^, UV detection at 210 nm. Data acquisition and processing were carried out using a LC-solution chromatography data system.

### 2.6. Thermodynamic analysis

Several parameters calculated by a van’t Hoff model, Gibbs free energy change (ΔG), enthalpy change (ΔH) and entropy change (ΔS), were used to evaluate the thermodynamic properties of the HPLC separation [31].

## 3. Results and discussion

### 3.1 Characterizations of UiO-66 analogues

**Fig 1:**
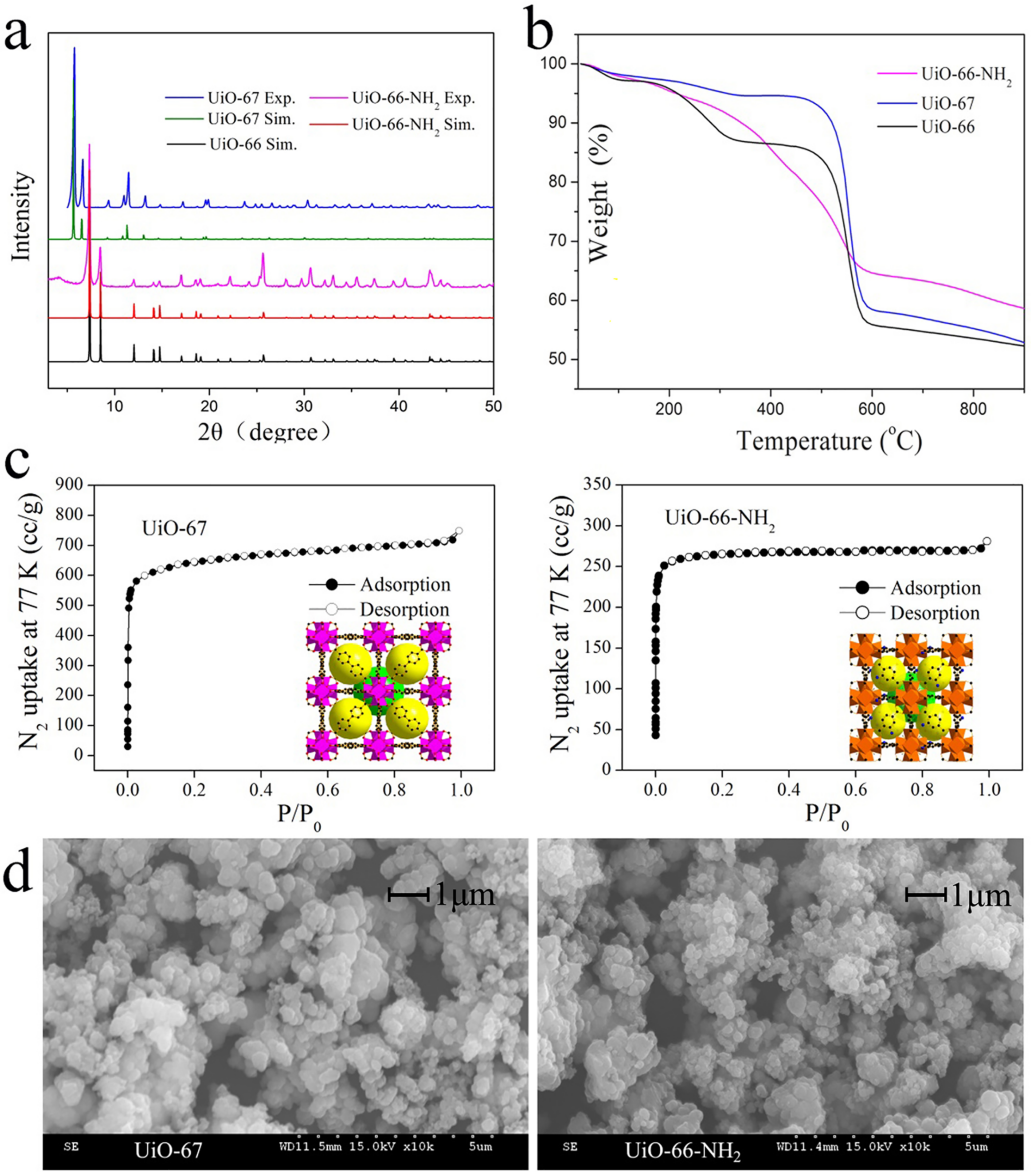
Characterizations of UiO-66 analogues: a XRD patterns; b TG curves; c N_2_ adsorption-desorption isotherms; d SEM images.

The UiO-66-NH_2_ is a product of amino-functionalized UiO-66 with–NH_2_ pointing into the pores. The UiO-67 is a product of linker-lengthened UiO-66 and has larger pore sizes (12 and 16 Å) compared to UiO-66 (8 and 11 Å). Physico-chemical properties of UiO-66 have been reported in our previous study [17]. Here, PXRD, TG, N_2_ adsorption-desorption and SEM experiments were further conducted to characterize the properties of UiO-66-NH_2_ and UiO-67. The experimental PXRD patterns of the UiO-66-NH_2_ and UiO-67 matched well with respectively simulated curves (from single-crystal structure data), indicating a successful preparation of UiO-66-NH_2_ and UiO-67 (Fig 1A). UiO-66-NH_2_ has very similar diffraction peaks with UiO-66 in the XRD pattern, suggesting that they are isostructural configurations. The UiO-66-NH_2_ has a relatively lower thermal stability than UiO-66, while UiO-67 has higher thermal stability than UiO-66 (Fig 1B). The results of N_2_ adsorption-desorption isotherms at 77 K indicated that UiO-66-NH_2_ and UiO-67 are typical microporous materials (Fig 1C). BET surface areas of the UiO-66-NH_2_ and UiO-67 are 1070 and 2180 m^2^·g^-1^, respectively. Compared to UiO-66 (BET surface area 1200 m^2^·g^-1^), UiO-66-NH_2_ possesses smaller surface area due to the presence of -NH_2_, While UiO-67 has nearly twice surface area due to the longer linker ligand 4,4’-biphenyl. In addition, the activated UiO-66-NH_2_ and UiO-67 have a relatively broad particle size distribution ranging from 150 to 450 nm (Fig 1D).

### 3.2 HPLC separation of SBs on UiO-66-NH_2_

#### 3.2.1 Effects of mobile phase on the separation

**Fig 2:**
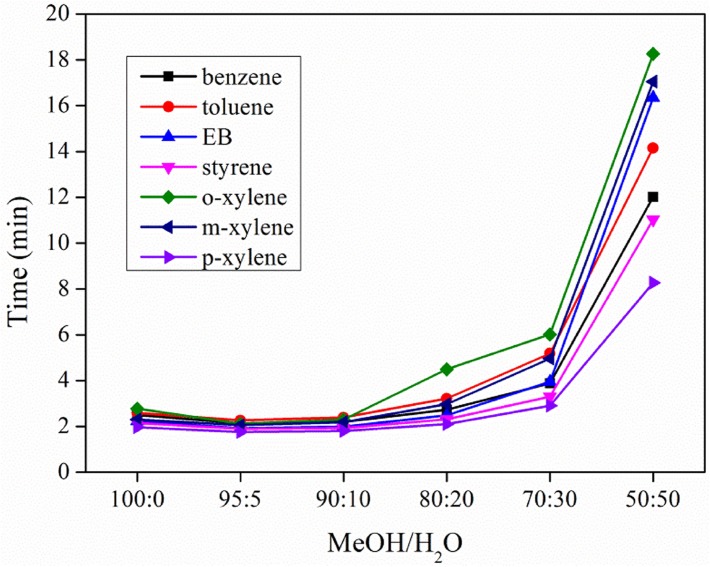
Effects of the proportion of MeOH/H_2_O on the retention times of SBs on UiO-66-NH_2_ packed column (5 cm long, 4.6 mm i.d.).

Mobile phases composed of MeOH and H_2_O at different ratios were used to determine the performance of UiO-66-NH_2_ in RP-HPLC separation of benzene and toluene, or ethylbenzene (EB), styrene and xylenes (Fig 2). It was found that the increase of water proportion in the mobile phases from 0% to 10% resulted in a less retention time of SBs on UiO-66-NH_2_ packed column. However, further increase of water resulted in the increase of retention time. The decreased retention time of SBs on UiO-66-NH_2_ column with increasing H_2_O from 0% to 10% in mobile phases is most likely due to the high affinity of the open-metal site in UiO-66-NH_2_ for lone pair of electrons in water molecule [32]. Water molecules in the mobile phase could occupy the open metal sites in UiO-66-NH_2_, reducing the interactions between SB analytes and UiO-66-NH_2_ and resulting in a short elution time. However, with the proportion of H_2_O increased from 10% to 50% in mobile phase, the neutral SB molecules had a poor solubility in the strong polar solvent based on the similar dissolve mutually theory and consequently resulted in a longer retention time on UiO-66-NH_2_ column.

**Fig 3:**
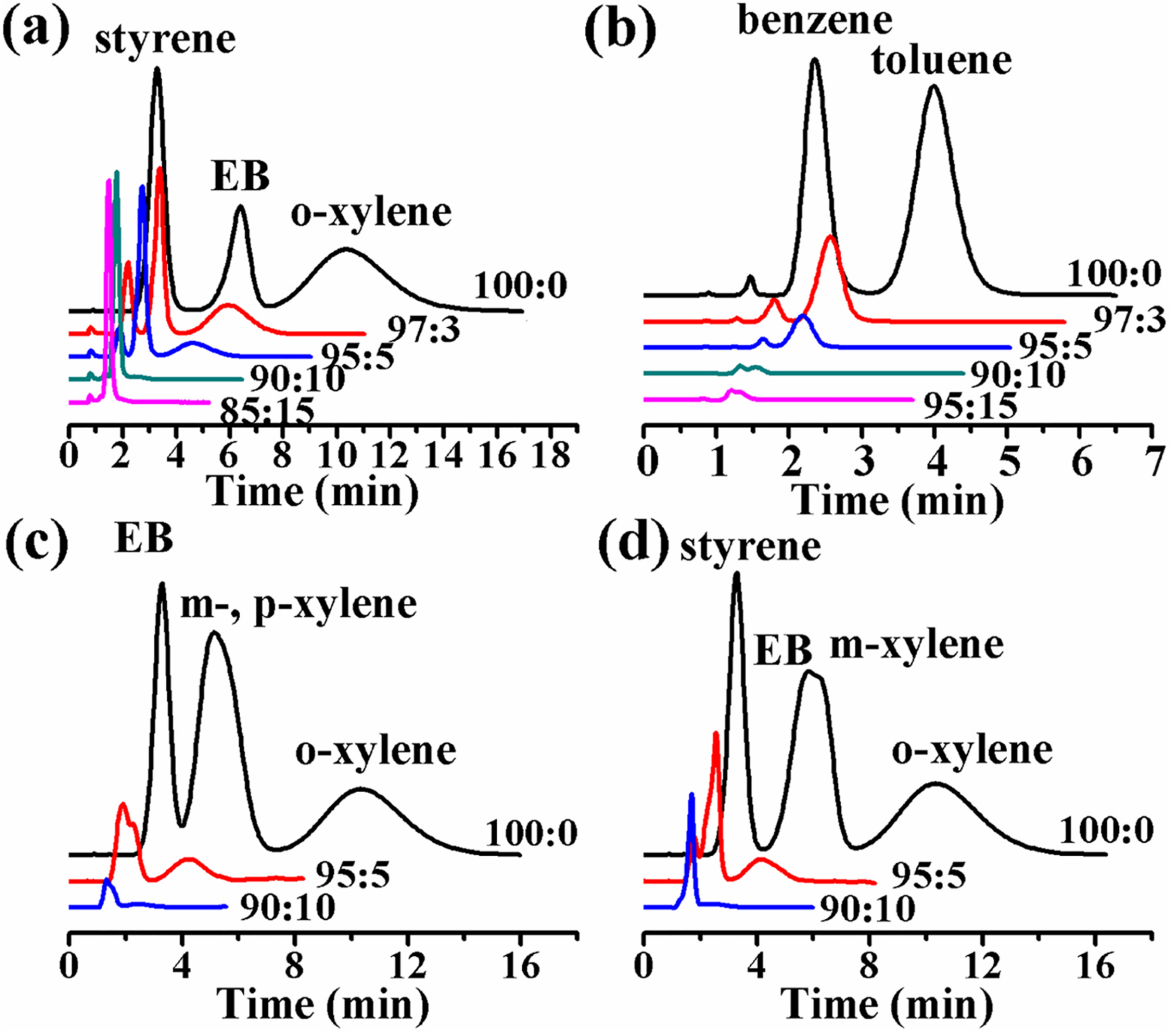
NP-HPLC separations of SBs on UiO-66-NH_2_ packed column (5 cm long, 4.6 mm i.d.): (a) styrene, EB and *o-*xylene; (b) benzene and toluene; (c) EB, *m-*, *p-* and *o-*xylene; (d) styrene, EB, *m-* and *o-*xylene. Conditions: mobile phase, n-hexane/DCM with different ratios in each separation; flow rate, 1.0 mL·min^-1^; UV detection at 210 nm; and column temperature 30°C.

Different ratios of the mixtures of at n-hexane and DCM were also used as mobile phases to investigate the performance of UiO-66-NH_2_ in NP-HPLC separation of SBs. When n-hexane alone was used as a mobile phase, the SB congeners were eluted in the way of long retention time, poor resolution and broad peak. Adding of DCM even in a little amount to n-hexane would greatly improve the resolution and dramatically decrease the retention time. This phenomenon might be explained by the competitive adsorption theory [33, 34]. When n-hexane/DCM (97:3) was used as mobile phase, baseline separations of EB, styrene and *o-*xylene, as well as benzene and toluene were achieved (Fig 3). However, further increase of DCM content in n-hexane/DCM mixtures would remarkably decrease resolution and shorten retention time. Particularly, 10% or more of DCM in n-hexane/DCM would lead to the co-elution of analytes.

#### 3.2.2 RP-HPLC separation of SBs on UiO-66-NH_2_

Compared to the performance of SBs on UiO-66 column [17], SBs on UiO-66-NH_2_ column could not be efficiently separated irrespective of the different retention times between SBs. The retention times of three xylene isomers on UiO-66-NH_2_ column followed the order *p*-xylene < *m*-xylene < *o*-xylene, which was the same to the order on UiO-66 column [17]. However, the retention times of the three xylene isomers on UiO-66-NH_2_ column were shorter than the corresponding retention times of each xylene isomer on UiO-66 column under the same MeOH/H_2_O. Since xylene isomers are neutral compounds, their adsorptions on UiO-66-NH_2_ and UiO-66 might be attributed to the action of van der Waals force [25]. Because of the partial blocking of pores by–NH_2_ groups, the surface area of UiO-66-NH_2_ (1070 m^2^∙g^-1^) was smaller than that of UiO-66 (1200 m^2^∙g^-1^), which led to weaker van der Waals force between xylenes and UiO-66-NH_2_ and resulted in faster elution (i.e., shorter retention times) of xylenes on UiO-66-NH_2_ column than on UiO-66 column.

#### 3.2.3 NP-HPLC separation of xylene isomers on UiO-66-NH_2_

On UiO-66-NH_2_ packed column, *o-*xylene could be separated from *p-* and *m-*xylene, while the separation of *m-* and *p-*xylene was failed. Similar to UiO-66, UiO-66-NH_2_ has a preferential affinity toward *o*-xylene. This might be explained in two aspects [17]. Firstly, *O*-xylene which has the largest molecular size among SBs had the strongest Van der Waals interaction with the UiO-66-NH_2_ framework [14], and consequently retained longer on the UiO-66-NH_2_ packed column than the other SBs did. Secondly, the two methyl groups in *o*-xylene had a strong interaction with carboxylate groups in UiO-66-NH_2_, which might also result in a longer retention of *o*-xylene on UiO-66-NH_2_ packed column [35].

Unlike the performance of *o*-xylene on UiO-66 column, the baseline separation of *m*-xylene and *p*-xylene failed on UiO-66-NH_2_ column [17]. This might be due to the lower porosity as the pores in UiO-66-NH_2_ could be partially blocked by–NH_2_. As a result, the surface area decreased and the interaction between xylenes and UiO-66-NH_2_ reduced_._ In addition, only one methyl group was available in *m*- and *p*-xylene and the methyl group could similarly interact with the carboxylate groups in UiO-66-NH_2_, which made *m-* and *p-*xylene be co-eluted from UiO-66-NH_2_ [35].

#### 3.2.4 NP-HPLC separation of EB and styrene on UiO-66-NH_2_

EB and styrene could be separated on UiO-66-NH_2_ packed column. And the retention time of styrene was shorter than EB. The retention order of EB and styrene on UiO-66-NH_2_ is opposite to that on UiO-66 [17] (Fig 3). The preference of UiO-66 for styrene over EB was observed on other terephthalic linked MOFs, such as MIL-47 and MIL-53 [36]. The less preference to styrene than EB of UiO-66-NH_2_ might be ascribed to its geometry configuration [37]. Styrene had a smaller molecule size (0.96×0.70×0.34 nm) than EB (0.95×0.67×0.53 nm), which enabled styrene to be easy to escape from the trapping of the small pores of UiO-66-NH_2_. Therefore, it was expected that the retention time of styrene on UiO-66-NH_2_ column was shorter than that of EB.

#### 3.2.5 Evaluation of separation performance on UiO-66-NH_2_

The reproducibility and selectivity of UiO-66-NH_2_ packed column for the NP-HPLC separation of SBs were evaluated using two blends: benzene and toluene, and EB, styrene and *o-*xylene (Fig 3, S1 Fig, S1 and S2 Tables). The relative standard deviations (RSD) of retention time (t_R_), peak area, peak height, and half peak width (W_1/2_) for five replicates ranged from 0.06 to 0.08%, 0.15 to 0.98%, 0.20 to 0.88%, and 0.17 to 0.40%, respectively. Therefore, it was concluded that the UiO-66-NH_2_ packed column gave good reproducibility for NP-HPLC separation of SBs. Also, it was found that there was a linear increase of the chromatographic peak area and peak height with the increase of mass of analytes (S2 Fig). However, the retention time and selectivity were independent of the mass of analytes (S2 Table). Nonetheless, the column efficiency generally decreased with the increase of analyte mass (S3 Fig).

### 3.3 HPLC separation of SBs and PAHs on UiO-67

#### 3.3.1 NP-HPLC separation of SBs on UiO-67

To evaluate the separation performance of UiO-67 as stationary phase, one-component injected analysis of SBs using different ratios of n-hexane/DCM (100:0, 95:5, 90:10, 85:15, 80:20, 70:30, 40:60, v/v) as mobile phases were investigated (S3 Table, S4 Fig). It was found that SB analytes almost coeluted within a short time (approximately 0.6 to 2.2 min) and displayed the expected insensitivity of retention time to mobile phase composition. On the contrary, the separations of SBs on UiO-66 packed column were successful in terms of the reverse shape selectivity, size selectivity and stacking effect as results of MOFs cages and van der Waals force [17]. The kinetic diameter of SBs (5.85–7.4 Å) is comparable to the sizes of UiO-66 cavities (8 to 11 Å) [19]. Therefore, SB molecules could enter the cavities of UiO-66 and interacted with cavity walls, resulting in an effective separation of SBs. However, the sizes of UiO-67 cavities (12 to 16 Å) are much bigger than the kinetic diameters of SBs [38]. SB molecules could pass through the cavities of UiO-67 through diffusion, enabling the equilibration and desorption of SBs on UiO-67 column to be easy and fast [13]. Therefore, it is proposed that the cavity sizes of MOFs made great contribution to the chromatographic separation of SBs.

#### 3.3.2 NP-HPLC separation of PAHs on UiO-67

In order to further explore the roles of pore size of UiO-66 analogues in chromatographic separation, NP-HPLC separation of large molecule PAHs on UiO-67 column were studied. Several PAHs with different numbers of rings, including naphthalene (two rings), fluorene, acenaphthene, anthracene and phenanthrene (three rings), pyrene and chrysene (four rings) were used. Also, different ratios of n-hexane/DCM were used as mobile phases. Based on the one-component injected experiments, two PAH mixtures: (1) naphthalene, anthracene and chrysene; and (2) naphthalene, phenanthrene and chrysene were used for NP-HPLC separation on the UiO-67 column.

**Fig 4:**
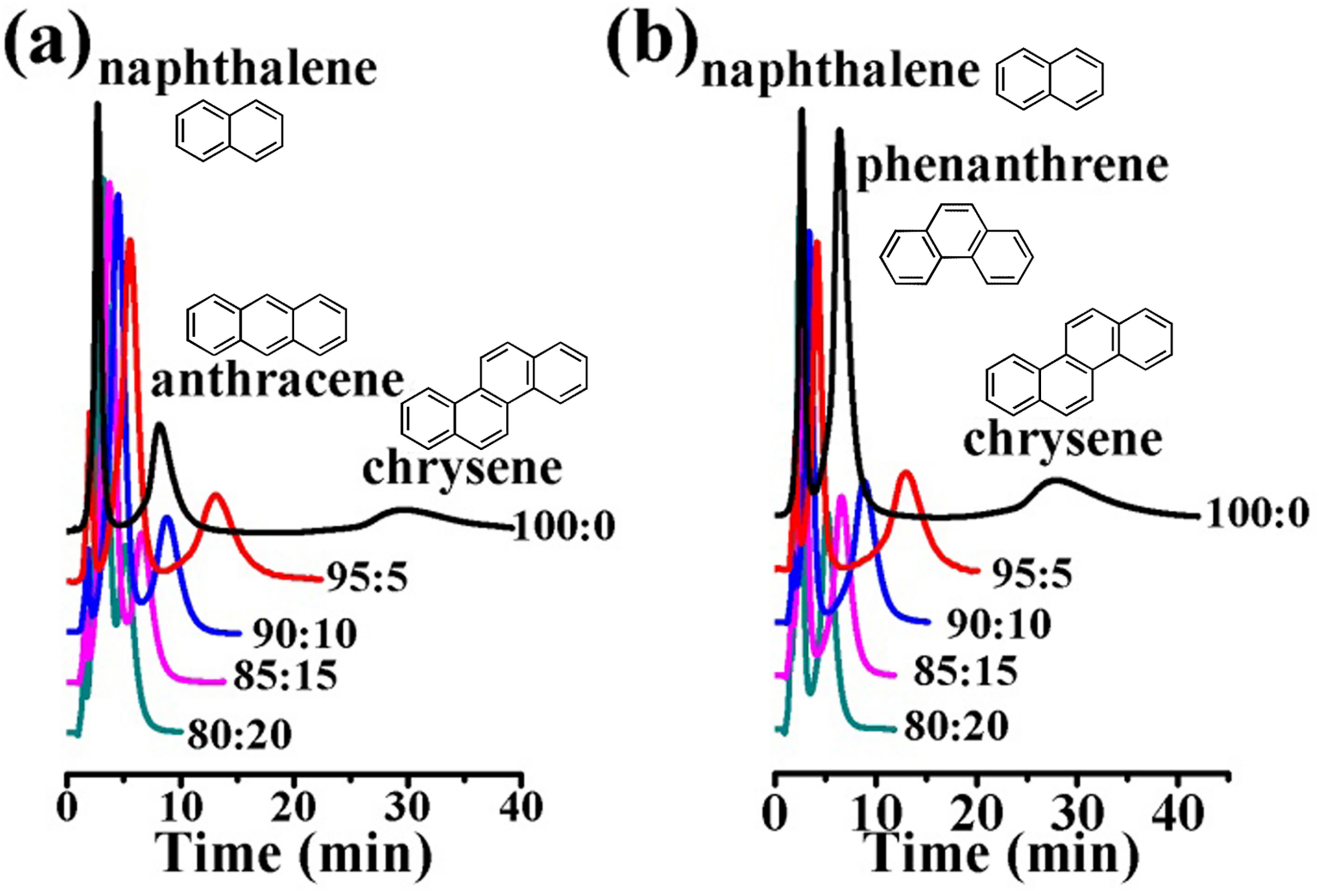
Chromatograms of PAHs on UiO-67 packed column (5 cm long, 4.6 mm i.d.): (a) naphthalene, anthracene, chrysene; (b) naphthalene, phenanthrene, chrysene. Conditions: mobile phase, n-hexane/DCM; flow rate, 1.0 mL·min^-1^; UV detection at 210 nm; column temperature, 30°C.

The ratio of n-hexane/DCM showed significant effect on NP-HPLC separation of PAHs on UiO-67 column (Fig 4). When n-hexane alone was used as mobile phase, the PAHs in both mixtures could generally be separated with long elution time needed and a poor peak pattern observed. Addition of 5% DCM to n-hexane (n-hexane/DCM = 95:5) remarkably improved the separation and baseline separations (R > 1.5) of PAHs in both mixtures, and the elution time of PAHs became shorter compared to that using n-hexane alone as mobile phase. However, further increase of DCM in n-hexane/DCM mixtures would lead to inefficient separation of PAHs, and even resulted in co-elution.

As shown in Fig 4, the retention order of PAHs on UiO-67 was as follows: PAHs with four rings > PAHs with three rings > PAHs with two rings. The π-π interactions between PAHs and aromatic framework walls of UiO-67 might be strong and dominantly important for the separation of PAHs. The more rings PAHs have, the stronger interactions between PAHs and the framework walls, which resulted in a longer retention time. It worth noting that inverse retention orders of PAHs on UiO-66 column (5 cm long, 4.6 mm i.d.) have been reported: PAHs with four rings < PAHs with three rings < PAHs with two rings [17]. This discrepancy might be ascribed to the size-exclusion effect. When UiO-66 was used as stationary phase to separate chrysene, fluorene and naphthalene, the PAHs with larger molecule sizes could be more likely to be excluded from the pores of UiO-66 and hence be eluted in shorter time.

### 3.4 Thermodynamics of HPLC separation on UiO-66 analogues

Thermodynamics of HPLC separation of analytes on UiO-66-NH_2_ and UiO-67 were investigated under different temperatures. The mixture of EB, styrene and *o-*xylene and the mixture of benzene and toluene were separated on UiO-66-NH_2_ column with temperatures ranged from 20°C to 60°C (S5 Fig), while the mixture of naphthalene, anthracene and chrysene and the mixture of naphthalene, phenanthrene and chrysene were separated on UiO-67 column with temperatures ranged from 20°C to 40°C (S6 Fig). As the column temperature increased, the retention time of analytes on either UiO-66-NH_2_ or UiO-67 decreased, indicating the involvement of the exothermic HPLC separation process. It is interesting that the selectivity of analytes on UiO-66-NH_2_ column slightly decreased as the temperature increased (S4 Table). However, the column temperature had no significant effect on the selectivity of analytes on UiO-67 column (S5 Table).

**Fig 5:**
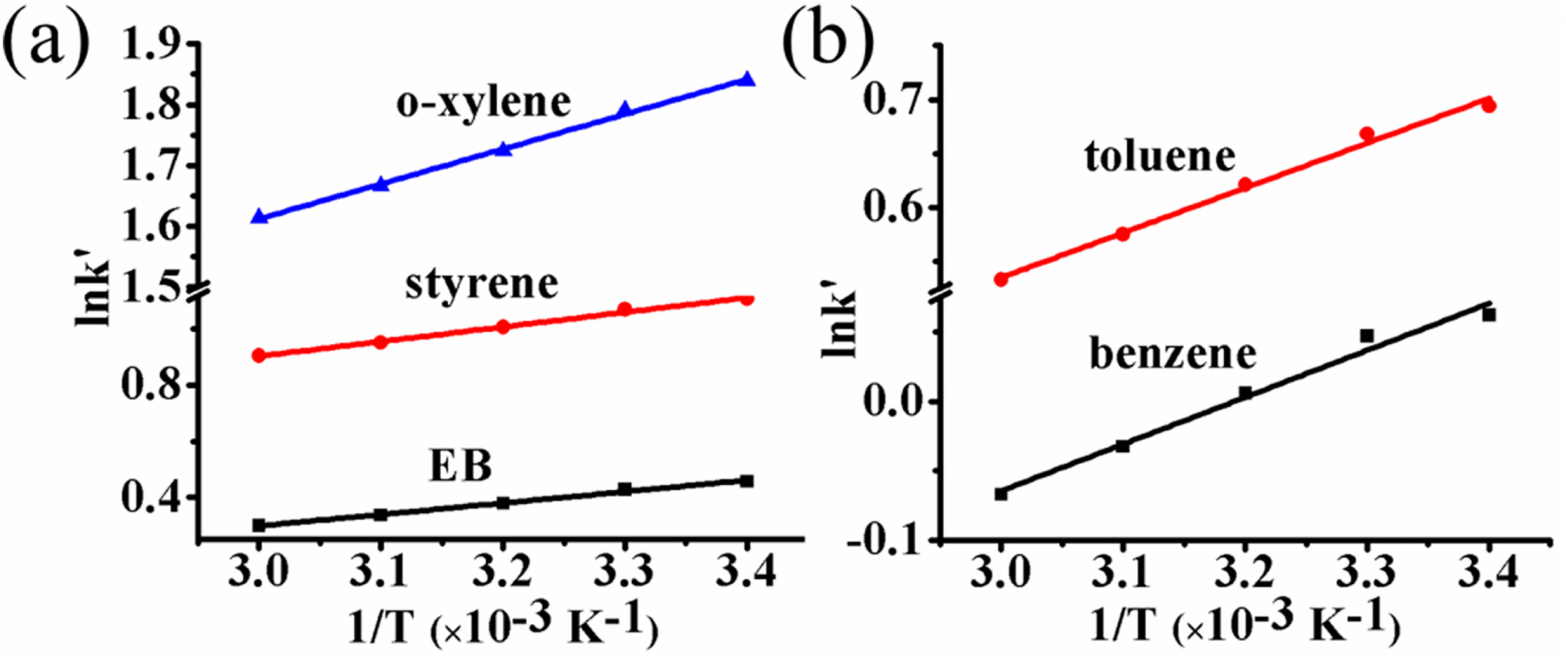
Van’t Hoff plots for analytes separated on UiO-66-NH_2_ packed column: (a) EB, styrene, *o-*xylene; (b) benzene and toluene. Conditions: mobile phase, n-hexane/DCM (97:3), flow rate, 1.0 mL·min^-1^; UV detection at 210 nm.

The van′t Hoff equation was used to explain the relationship between retention factor k' and column temperature [31]. A good linearity between lnk' and 1/T suggested that there was no change in the mechanism for the NP-HPLC separation of SBs on UiO-66-NH_2_ at the temperature range of 20 to 60°C (Fig 5). The thermodynamic parameters obtained from the van’t Hoff equation for the transfer of analytes from mobile phase to the UiO-66-NH_2_ stationary phase were summarized and presented in Table 1. The negative values of ΔG indicated that the analytes transfer processes on UiO-66-NH_2_ were thermodynamically spontaneous. The smaller negative values of ΔG indicate the more favorable transfer of the analytes from the mobile phase to the stationary phase and thus the longer retention time on the stationary phase. In addition, comparative analysis of ΔH and ΔS on different analytes showed that the values of ΔH of different analytes were comparable but the values of ΔS varied considerably. Thus, the NP-HPLC separations of these analytes on UiO-66-NH_2_ stationary phase were controlled only by positive ΔS.

**Table 1:** Values of ΔH, ΔS, ΔG and R^2^ for the NP-HPLC separation of SBs on UiO-66-NH_2_ packed column.

Analyte	ΔH (kJ·mol^-1^)	ΔS (J·mol^-1^·k^-1^)	ΔG (kJ·mol^-1^)	R^2^
**benzene**	-2.81 ±0.21	5.39 ±0.68	-4.44 ± 0.21	0.97768
**toluene**	-3.45 ±0.18	8.45 ±0.57	-6.01 ± 0.18	0.98953
**EB**	-3.39 ±0.15	6.65 ±0.47	-5.41 ± 0.15	0.99248
**styrene**	-4.35 ±0.19	8.82 ± 0.62	-7.02 ± 0.19	0.99223
***o-*xylene**	-4.77 ±0.13	13.43± 0.41	-8.84 ± 0.13	0.99716

## 4. Conclusions

The performances of UiO-66-NH_2_ and UiO-67 packed columns for HPLC separation of SBs and PAHs were evaluated in the present study. Parameters influencing HPLC separation, such as mobile phase composition, analyte mass, and column temperature were determined. Although not all analytes could be efficiently separated on UiO-66-NH_2_ column especially for RP-HPLC separation, the efficient separation of EB, styrene and *o-*xylene is applicable, suggesting a potential application of UiO-66-NH_2_ as stationary phase in NP-HPLC. To some extent, UiO-67 can be effectively used as stationary phase in NP-HPLC separation of molecules with relatively large diameter. Additionally, the functional groups and pore size of MOFs play important roles in the separation of organics on the HPLC instrument coupling with MOFs packed column.

## Supporting information

S1 Fig NP-HPLC chromatograms of styrene, EB, *o*-xylene, benzene and toluene on UiO-66-NH_2_ packed column for five repeat separations. (TIF)

S2 Fig Effects of SBs mass on peak area and peak height. (TIF)

S3 Fig Effects of SBs mass on column efficiency. (TIF)

S4 Fig Chromatogram of SBs on UiO-67 packed column. (TIF)

S5 Fig Chromatographic separations of SBs on UiO-66-NH_2_ packed column at different temperatures ranged from 20°C to 60°C. (TIF)

S6 Fig Chromatographic separations of PAHs on UiO-67 packed column. (TIF)

S1 Table Precision for five repeat NP-HPLC separations of SBs on UiO-66-NH_2_ packed column. (DOCX)

S2 Table Selectivity of SBs on the UiO-66-NH_2_ packed column with different injection masses. (DOCX)

S3 Table The retention times of SBs using different ratios of n-hexane/DCM as mobile phases on UiO-67 packed column. (DOCX)

S4 Table Selectivity of SBs at different temperatures on UiO-66-NH_2_ packed column in NP-HPLC process. (DOCX)

S5 Table Selectivity of PAHs at different temperatures on UiO-67 packed column in NP-PHLC process. (DOCX)
