# Supplementary figures and images for: Preparation, characterization, and performance evaluation of UiO-66 analogues as stationary phase in HPLC for the separation of substituted benzenes and polycyclic aromatic hydrocarbons
Source: PLoS One. 2017 Jun 5;12(6):e0178513. doi: 10.1371/journal.pone.0178513 (PMC5459429; doi:10.1371/journal.pone.0178513)

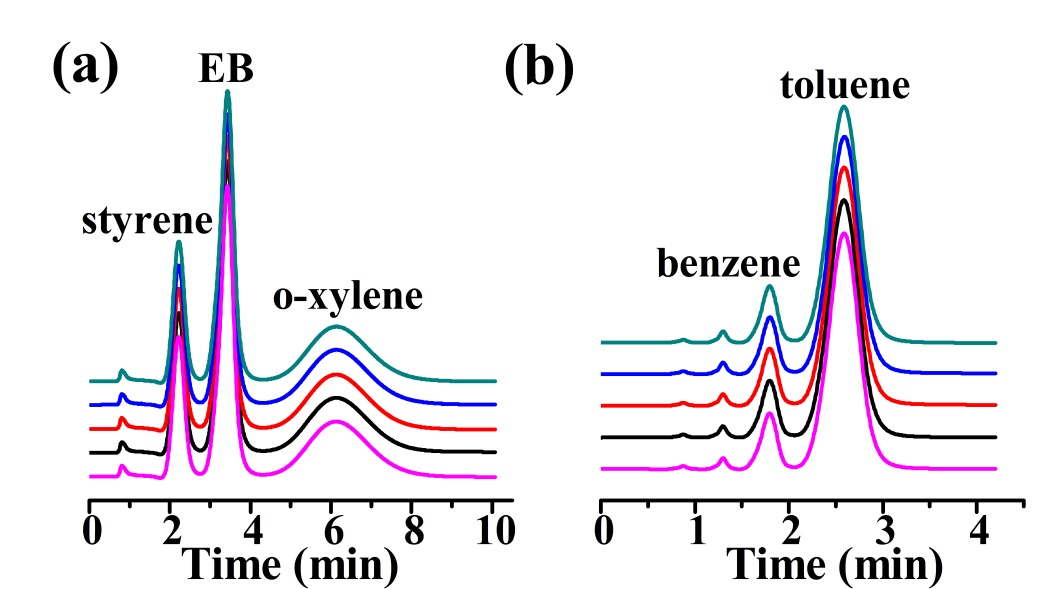

Supplement: S1 Fig — (TIF) [file pone.0178513.s001.tif]

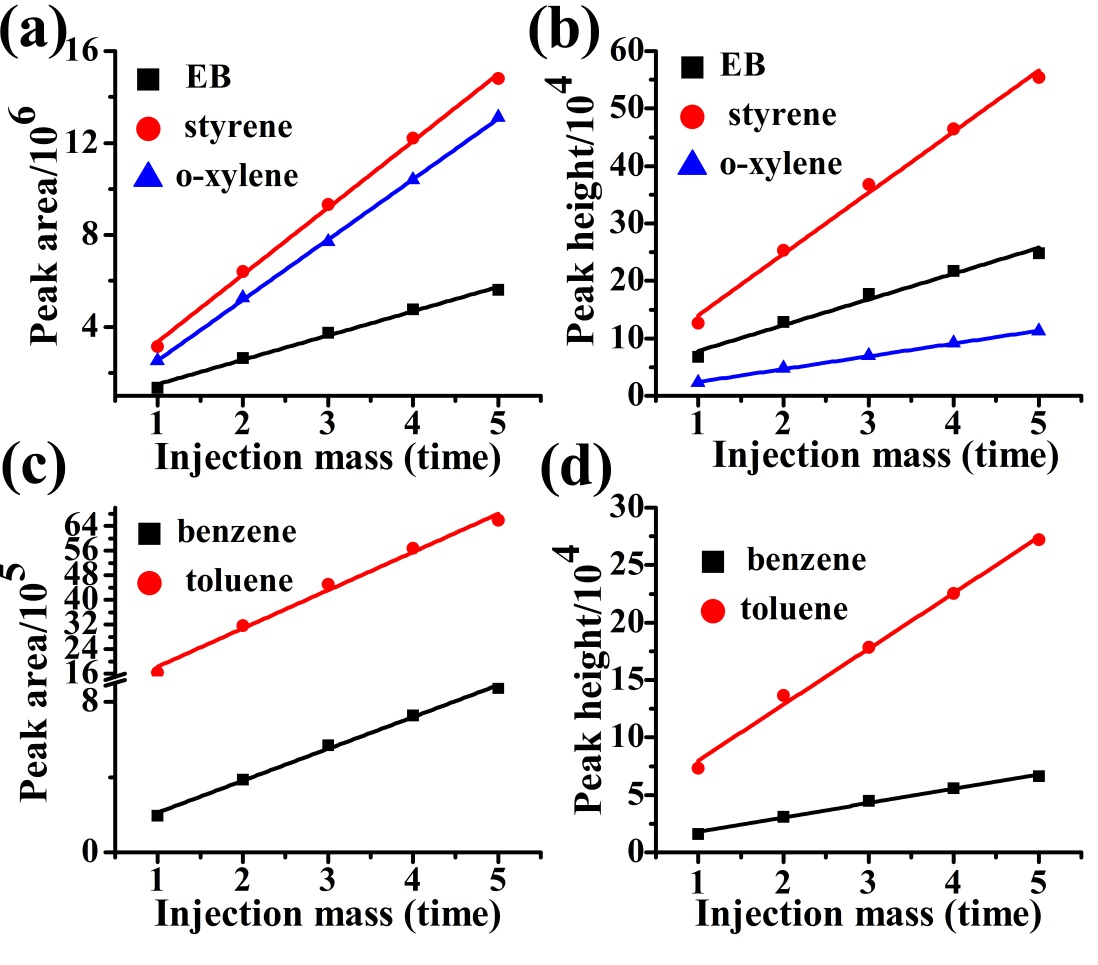

Supplement: S2 Fig — (TIF) [file pone.0178513.s002.tif]

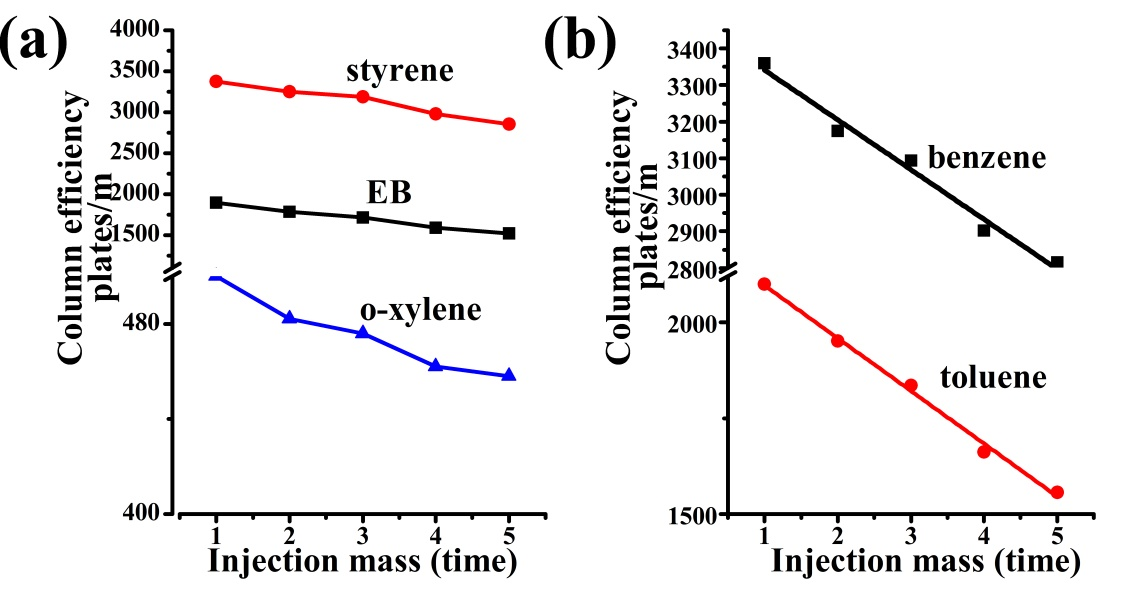

Supplement: S3 Fig — (TIF) [file pone.0178513.s003.tif]

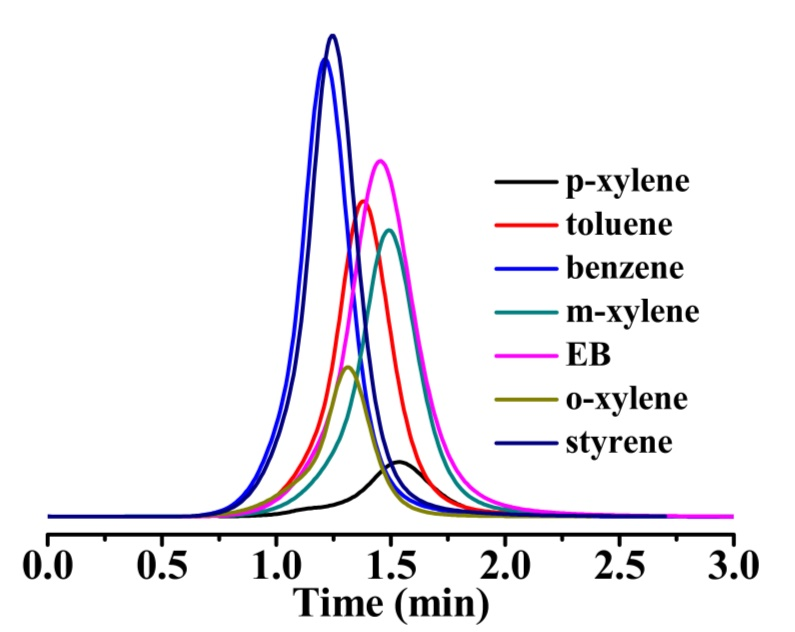

Supplement: S4 Fig — (TIF) [file pone.0178513.s004.tif]

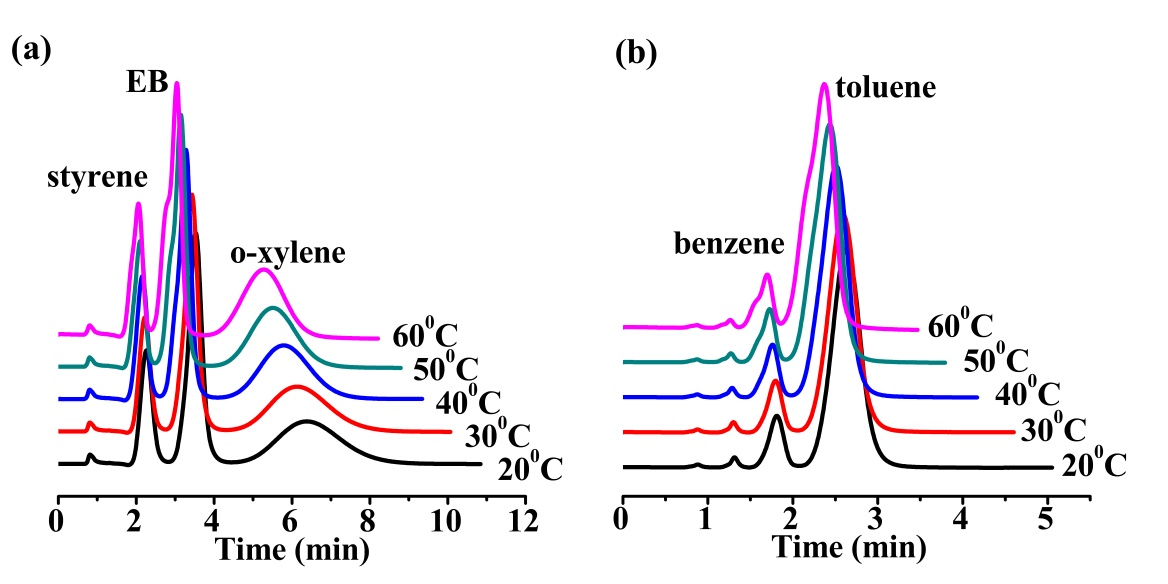

Supplement: S5 Fig — (TIF) [file pone.0178513.s005.tif]

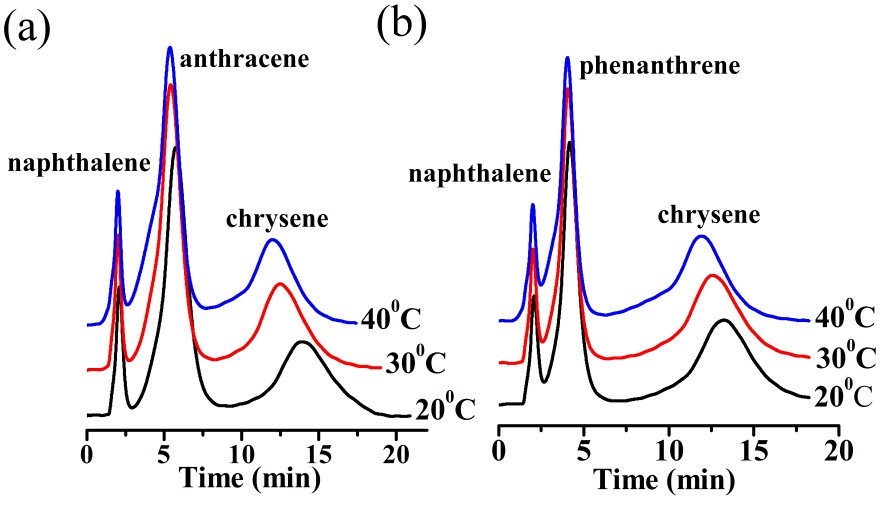

Supplement: S6 Fig — (TIF) [file pone.0178513.s006.tif]
