# Supplementary material for: Preparation, characterization, and performance evaluation of UiO-66 analogues as stationary phase in HPLC for the separation of substituted benzenes and polycyclic aromatic hydrocarbons
Source: PLoS One. 2017 Jun 5;12(6):e0178513. doi: 10.1371/journal.pone.0178513 (PMC5459429; doi:10.1371/journal.pone.0178513)
Supplement: S1 Table — (DOCX) [file pone.0178513.s007.docx]

**S1 Table. Precision for five repeat separations of SBs on UiO-66-NH_2_ packed column used in NP-HPLC process.**

| **Analyte** | **RSD (%) (n=5)** | | | |
| --- | --- | --- | --- | --- |
|  | **t_R_** | **Peak area** | **Peak height** | **W_1/2_** |
| **benzene** | 0.07 | 0.87 | 0.88 | 0.29 |
| **toluene** | 0.08 | 0.65 | 0.38 | 0.36 |
| **EB** | 0.06 | 0.16 | 0.20 | 0.17 |
| **styrene** | 0.06 | 0.15 | 0.34 | 0.25 |
| ***o-*xylene** | 0.06 | 0.98 | 0.45 | 0.40 |
