# Supplementary material for: Preparation, characterization, and performance evaluation of UiO-66 analogues as stationary phase in HPLC for the separation of substituted benzenes and polycyclic aromatic hydrocarbons
Source: PLoS One. 2017 Jun 5;12(6):e0178513. doi: 10.1371/journal.pone.0178513 (PMC5459429; doi:10.1371/journal.pone.0178513)
Supplement: S2 Table — (DOCX) [file pone.0178513.s008.docx]

**S2 Table. Selectivity of SBs with different injection masses in the NP-HPLC separation process using UiO-66-NH_2_ packed column.**

| **Injection mass/time** | **Selectivity (α)** | | |
| --- | --- | --- | --- |
|  | **EB, styrene, *o-*xylene** | | **benzene, toluene** |
|  | **EB/ styrene** | **styrene/*o-*xylene** | **benzene/toluene** |
| **1** | 1.55 | 1.79 | 1.44 |
| **2** | 1.55 | 1.79 | 1.44 |
| **3** | 1.55 | 1.79 | 1.44 |
| **4** | 1.55 | 1.79 | 1.44 |
| **5** | 1.54 | 1.79 | 1.44 |
