# Supplementary material for: Preparation, characterization, and performance evaluation of UiO-66 analogues as stationary phase in HPLC for the separation of substituted benzenes and polycyclic aromatic hydrocarbons
Source: PLoS One. 2017 Jun 5;12(6):e0178513. doi: 10.1371/journal.pone.0178513 (PMC5459429; doi:10.1371/journal.pone.0178513)
Supplement: S3 Table — (DOCX) [file pone.0178513.s009.docx]

**S3 Table. The retention times of SBs at different ratios of n-hexane/DCM as mobile phases on UiO-67 packed column in NP-HPLC process.**

| **Analyte** | **Mobile phase (n-hexane/DCM)** | | | | | | |
| --- | --- | --- | --- | --- | --- | --- | --- |
|  | **100:0** | **95:5** | **90:10** | **85:15** | **80:20** | **70:30** | **40:60** |
| **benzene** | 1.532 | 1.482 | 1.390 | 1.307 | 1.226 | 1.107 | 0.911 |
| **toluene** | 1.376 | 1.329 | 1.235 | 1.187 | 1.115 | 1.013 | 0.850 |
| **EB** | 1.209 | 1.188 | 1.105 | 1.070 | 1.012 | 0.920 | 0.776 |
| **styrene** | 1.488 | 1.426 | 1.309 | 1.236 | 1.154 | 1.033 | 0.837 |
| ***o-*xylene** | 1.451 | 1.376 | 1.266 | 1.204 | 1.127 | 1.017 | 0.843 |
| ***m-*xylene** | 1.108 | 1.096 | 1.021 | 0.987 | 0.944 | 0.875 | 0.749 |
| ***p-*xylene** | 1.241 | 1.230 | 1.147 | 1.107 | 1.046 | 0.951 | 0.806 |
