# Supplementary material for: Preparation, characterization, and performance evaluation of UiO-66 analogues as stationary phase in HPLC for the separation of substituted benzenes and polycyclic aromatic hydrocarbons
Source: PLoS One. 2017 Jun 5;12(6):e0178513. doi: 10.1371/journal.pone.0178513 (PMC5459429; doi:10.1371/journal.pone.0178513)
Supplement: S4 Table — (DOCX) [file pone.0178513.s010.docx]

**S4 Table. Selectivity of SBs at different temperatures on UiO-66-NH_2_ packed column in NP-HPLC process.**

| **T/°C** | **Selectivity (α)** | | |
| --- | --- | --- | --- |
|  | **EB, styrene, *o-*xylene** | | **benzene, toluene** |
|  | **EB/styrene** | **styrene/*o-*xylene** | **benzene/styrene** |
| **20** | 1.56 | 1.81 | 1.45 |
| **30** | 1.55 | 1.79 | 1.44 |
| **40** | 1.52 | 1.77 | 1.43 |
| **50** | 1.50 | 1.75 | 1.41 |
| **60** | 1.48 | 1.74 | 1.40 |
