# Supplementary material for: Preparation, characterization, and performance evaluation of UiO-66 analogues as stationary phase in HPLC for the separation of substituted benzenes and polycyclic aromatic hydrocarbons
Source: PLoS One. 2017 Jun 5;12(6):e0178513. doi: 10.1371/journal.pone.0178513 (PMC5459429; doi:10.1371/journal.pone.0178513)
Supplement: S5 Table — (DOCX) [file pone.0178513.s011.docx]

**S5 Table. Selectivity of PAHs at different temperatures on UiO-67 packed column in NP-PHLC process.**

| T/°C | Selectivity (α) | | | |
| --- | --- | --- | --- | --- |
|  | naphthalene (NAP),  anthracene (ANT),  chrysene (CHR) | | naphthalene (NAP),  phenanthrene (PHE),  chrysene (CHR) | |
|  | NAP/ANT | ANT/CHR | NAP/PHE | PHE/CHR |
| 20 | 2.126 | 1.901 | 1.680 | 2.463 |
| 30 | 2.115 | 1.771 | 1.786 | 2.456 |
| 40 | 2.129 | 1.721 | 1.731 | 2.389 |
